# Molecular Signature of Biological Aggressiveness in Clear Cell Sarcoma of the Kidney (CCSK)

**DOI:** 10.3390/ijms24043743

**Published:** 2023-02-13

**Authors:** Michele Fiore, Alberto Taddia, Valentina Indio, Salvatore Nicola Bertuccio, Daria Messelodi, Salvatore Serravalle, Jessica Bandini, Filippo Spreafico, Daniela Perotti, Paola Collini, Andrea Di Cataldo, Gianandrea Pasquinelli, Francesca Chiarini, Maura Fois, Fraia Melchionda, Andrea Pession, Annalisa Astolfi

**Affiliations:** 1 Orthopaedics and Traumatology Unit, IRCCS Azienda Ospedaliero-Universitaria di Bologna, 40138 Bologna, Italy; 2 Department of Medical and Surgical Sciences, DIMEC, University of Bologna, 40138 Bologna, Italy; 3 Department of Veterinary Medical Sciences, University of Bologna, Ozzano dell’Emilia, 40064 Bologna, Italy; 4 Pediatric Oncology and Hematology “Lalla Seràgnoli”, IRCCS Azienda Ospedaliero-Universitaria di Bologna, 40138 Bologna, Italy; 5 Pediatric Oncology Unit, Department of Medical Oncology and Hematology, Fondazione IRCCS Istituto Nazionale dei Tumori, 20133 Milan, Italy; 6 Molecular Bases of Genetic Risk and Genetic Testing Unit, Department of Experimental Oncology, Fondazione IRCCS Istituto Nazionale dei Tumori, 20133 Milan, Italy; 7 Soft Tissue Tumor Pathology Unit, Advanced Diagnostics Department, Fondazione IRCCS Istituto Nazionale Dei Tumori, 20133 Milan, Italy; 8 Department of Clinical and Experimental Medicine, Unit of Pediatric Hematology and Oncology, University of Catania, 95124 Catania, Italy; 9 Department of Biomedical, Metabolic and Neural Sciences, University of Modena and Reggio Emilia, 41125 Modena, Italy; 10 Division of Pediatrics, IRCCS Azienda Ospedaliero-Universitaria di Bologna, 40138 Bologna, Italy

**Keywords:** CCSK, FGF3, BCOR, internal tandem duplication

## Abstract

Clear cell sarcoma of the kidney (CCSK) is a rare pediatric renal tumor with a worse prognosis than Wilms’ tumor. Although recently, BCOR internal tandem duplication (ITD) has been found as a driver mutation in more than 80% of cases, a deep molecular characterization of this tumor is still lacking, as well as its correlation with the clinical course. The aim of this study was to investigate the differential molecular signature between metastatic and localized BCOR-ITD-positive CCSK at diagnosis. Whole-exome sequencing (WES) and whole-transcriptome sequencing (WTS) were performed on six localized and three metastatic BCOR-ITD-positive CCSKs, confirming that this tumor carries a low mutational burden. No significant recurrences of somatic or germline mutations other than BCOR-ITD were identified among the evaluated samples. Supervised analysis of gene expression data showed enrichment of hundreds of genes, with a significant overrepresentation of the MAPK signaling pathway in metastatic cases (*p* < 0.0001). Within the molecular signature of metastatic CCSK, five genes were highly and significantly over-expressed: FGF3, VEGFA, SPP1, ADM, and JUND. The role of FGF3 in the acquisition of a more aggressive phenotype was investigated in a cell model system obtained by introducing the ITD into the last exon of BCOR by Crispr/Cas9 gene editing of the HEK-293 cell line. Treatment with FGF3 of BCOR-ITD HEK-293 cell line induced a significant increase in cell migration versus both untreated and scramble cell clone. The identification of over-expressed genes in metastatic CCSKs, with a particular focus on FGF3, could offer new prognostic and therapeutic targets in more aggressive cases.

## 1. Introduction

Clear cell sarcoma of the kidney (CCSK) is a rare pediatric renal tumor that represents 3–5% of all childhood renal tumors. It is the second most common malignant neoplasia of the kidney after Wilms’ tumor (WT) in the 0–14 age range [1]. Average age at onset is 36 months, ranging from rare pre-natal cases [1,2,3,4,5] to anecdotal cases in adults [1,6,7,8,9]. The incidence is twice as high in males than in females [10,11,12]. Unlike WT, CCSK does not appear to be associated with predisposing syndromes or to occur in individuals with germline genetic mutations [10].

Although CCSK is apparently a genetically stable tumor, two mutually exclusive genetic events have been systematically reported: different *BCOR* gene ITDs on the exon 16 in more than 80% of cases [13,14,15,16,17] and the *t* (10;17) involving the YWHAE-NUTM2 fusion in up to 12% of cases [13,14,15,16,17,18,19,20].

The *BCOR* (BCL6 corepressor) gene is located on chromosome Xp11.4 [21,22,23]. Its location on the X chromosome is probably the reason for the higher incidence of CCSK in males, due to the fact that in cells from males, the single X chromosome bearing the anomaly is active, whereas in cells from females, the X-inactivation halves the number of cells potentially initiated to tumorigenesis. *BCOR* encodes for a ubiquitously expressed nuclear protein that is essential for the constitution of one of the six currently described noncanonical variants of PRC1, PRC1.1 (Polycomb Repressive Complex type 1) [24,25,26,27], which epigenetically regulates the transcription of several genes involved in early embryonic development, mesenchymal stem cell function, and hemopoiesis [23,28]. Therefore, it can be assumed that the presence of the BCOR-ITDs leads to a deregulation of PRC activity with oncogenic significance in sensitive cells. While germline *BCOR* mutations are responsible for the X-linked oculo-facio-cardio-dental (OFCD) syndrome, somatic alterations were detected in various tumors, including sarcomas, Central Nervous System (CNS) tumors, hemo-lymphopoietic system tumors, and thymomas [23].

Regarding the transcriptional profile of CCSKs, up-regulation of the Sonic Hedgehog pathway, neural differentiation, and Akt/mTOR pathway and down-regulation of genes involved in focal adhesion processes were found [16,29,30,31,32,33,34]. In addition, EGFR [32,34,35,36], FGF9/13/19 [34], CCND1 (Cyclin-D1) [34,37,38], VEGFA [34,35,39,40], BCOR [14,15,41], and EZH2 [29] were found to be over-expressed. Immunohistochemically, CCSKs are strongly positive for vimentin [42,43], NGFR [44], and Cyclin-D1 [37,38,45,46].

CCSK is a more aggressive entity than WT. CCSK metastasizes (especially to lymph node and bone) and recurs more frequently, sometimes with typical late CNS metastases. Outcome was markedly affected by the improvement in chemo-radiotherapy protocols, with a current 5-year overall survival rate of 86% and a 5-year event-free survival rate of 78% [47,48]. Relapses occur in about 15% of the patients, with a 5-year event-free survival after relapse of 18% and a 5-year overall survival of 26% [6].

The aim of this study was to investigate the possibility of a differential molecular signature between metastatic and localized BCOR-ITD-positive CCSKs at the diagnosis, which could aid in the identification of more aggressive tumor entities.

## 2. Results

The series consisted of nine patients affected by CCSK and enrolled in the TW-2003 AIEOP (Associazione Italiana Ematologia Oncologia Pediatrica) protocol, whose clinical features are summarized in Table 1.

For all CCSKs, the primary tumor sample was analyzed, and the presence of BCOR-ITD was confirmed by PCR and Sanger sequencing. Three different BCOR-ITDs were identified (Figure S1): ITD-1 (c.5136_5225dup) was found in CCSK 2-4-7, ITD-2 (c.5099_5212dup) in CCSK 8, and ITD-3 (c.5171_5266dup) in CCSK 1-3-5-7-9 (Table 2).

The analysis of the mutational load of the single CCSK samples, in line with previous reports, confirmed CCSK as a tumor with a low mutational burden. Specifically, the mutational load of the nine cases varied between a maximum of 0.4 mutations/Mb for CCSK 1 and a minimum of 0.07 mutations/Mb for CCSK 5, with an average of 0.21 mutations/Mb. The comparison between the average mutational load of CCSK of this series and that of several tumors, previously described in the literature [49], places CCSK among the tumors less affected by somatic mutations, with a mutational load 1000 times lower than that of some melanomas. No significant recurrences of somatic or germinal mutations were identified among the evaluated samples.

The unsupervised analysis of whole-transcriptome sequencing (WTS) data on fresh frozen samples did not reveal any clear segregation of the global expression profiles of the cases with metastatic onset and the localized ones (Figure S2), in line with previous evidence [29]. Conversely, an evident clustering of samples based on the type of ITD carried was found (Figure S2), with a clear segregation between the group of three CCSKs characterized by ITD-1 and that of four CCSKs characterized by ITD-3 on the second principal component. This result was confirmed also by unsupervised hierarchical clustering.

**Figure 1:**
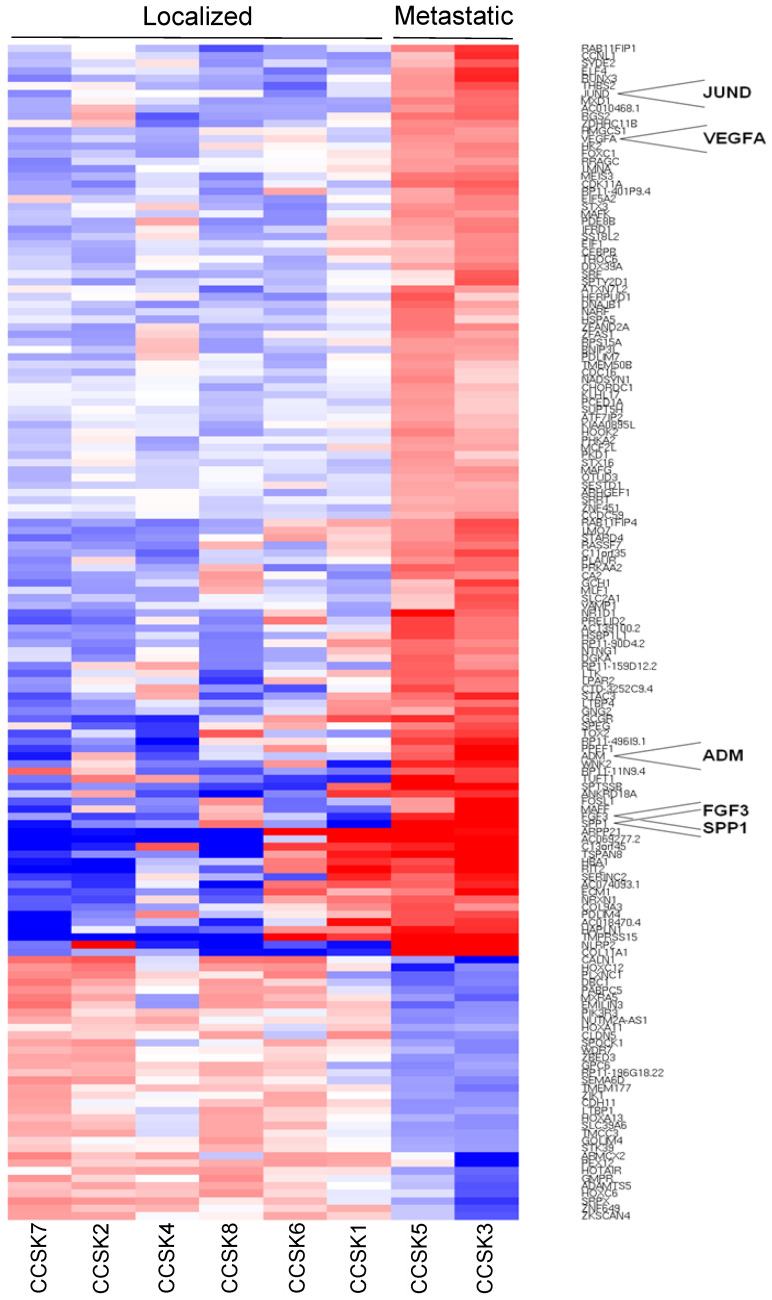
Hierarchical clustering of the 156 genes differentially expressed between metastatic and localized CCSK (121 up-regulated in metastatic CCSKs and 35 down-regulated; *p* < 0.01). The genes validated and discussed in study are highlighted.

**Figure 2:**
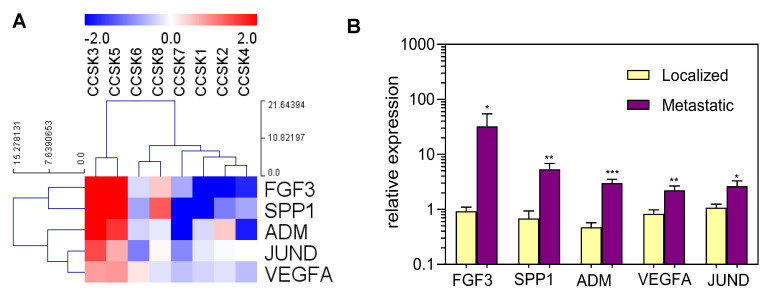
(**A**) Heatmap representation of selected differentially expressed genes between metastatic (CCSK3 and CCSK5) and localized samples as measured by WTS. (**B**) Validation of differentially expressed genes by quantitative PCR (*, *p* < 0.05; **, *p* < 0.01; ***, *p* < 0.001).

The supervised analysis of the expression profile of metastatic cases compared to localized ones identified 783 genes differentially expressed with a *p*-value < 0.05 and 156 with a *p*-value < 0.01 (Figure 1). The pathway enrichment analysis identified several protein-coding genes associated with “MAPK signaling pathway” significantly up-regulated in metastatic cases (11 genes; adj-*p* < 0.0001). The functional analysis of the molecular signature of metastatic cases highlighted the statistically significant over-expression of five genes: FGF3 (*p* = 0.0006), VEGFA (*p* = 0.0009), SPP1 (*p* = 0.0003), ADM (*p* = 0.0009), and JUND (*p* = 0.004) (Figure 2A).

Data from WTS were validated by quantitative PCR (Figure 2B), yielding results that confirmed the over-expression of the five genes. Moreover, FGF3 and VEGFA were also significantly over-expressed compared to five cases of WT by quantitative PCR (Figure S3).

**Figure 3:**
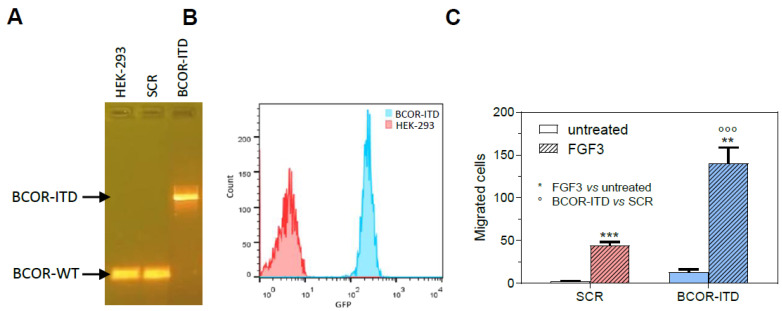
(**A**) PCR detection of BCOR-ITD construct integrated in the last exon of BCOR in HEK BCOR-ITD and not the parental HEK-293 cell line or scramble clone (SCR). (**B**) Flow cytometry detection of GFP fluorescence in BCOR-ITD and not in HEK-293 parental cell line. (**C**) Migration induced by FGF3 10 ng/mL in BCOR-ITD and SCR clones with respect to untreated cells (**, *p* < 0.01; ***/°°°, *p* < 0.001).

Since *FGF3* was by far the most over-expressed gene in metastatic CCSK, it is likely that its function is causally connected to the acquisition of a more aggressive phenotype. We therefore generated a disease model of CCSK by gene editing in the HEK-293 cell line, using as the donor vector for HDR recombination a pGL3 basic vector with the insertion of the ITD upstream of a GFP sequence, in frame with the last exon of BCOR (Figure S4). Limiting dilution cloning of the edited cell pool identified a clone (HEK BCOR-ITD) that had integrated the ITD construct in homozygosis (being the clone of a diploid female cell line), while the parental cell line and a scramble clone (SCR) were wild type at the last exon of BCOR (Figure 3A).

BCOR ITD was expressed also at the protein level, as shown by flow cytometry detection of GFP in the BCOR-ITD clone and not in the scramble clone (Figure 3B), being GFP in frame with the last exon of BCOR in the donor vector, and by specific BCOR antibody staining that showed a two-fold increase in BCOR-ITD clone with respect to the parental cell line (Figure S5).

The functional role of FGF3 in this cell model reproducing the oncogenic hit of CCSK was investigated analyzing cell growth and migration upon FGF3 treatment (Figure S6). While FGF3 was not active in inducing HEK BCOR-ITD growth advantage over SCR and the parental cell line, it was responsible for a specific and significant increase in cell migration over the untreated cell lines, with the highest number of migrated cells displayed by the BCOR-ITD clone treated with FGF3 (Figure 3C).

## 3. Discussion

This study confirmed that BCOR-ITD-positive CCSKs are a cytogenetically stable tumor with few genomic alterations and without a specific mutation profile shared by metastatic cases. In fact, no specific recurrent mutations were detected among metastatic CCSKs that would therefore potentially correlate with a greater biological aggressiveness.

For CCSK, in accordance with the lack of known bilateral synchronous presentations, neither pathogenic germinal mutations nor associated syndromes have yet been described. In line with this evidence, WES analysis did not evidence specific germline mutations already known to be causally associated with cancer predisposition.

The unsupervised analysis of the global expression profiles showed no clear segregation between metastatic onset and localized cases, while a clear clustering of samples was found based on the type of ITD, with a significant segregation between the group carrying ITD-1 and the group harboring ITD-3. This would suggest that different ITDs could affect in different ways the functionality of BCOR and consequently the transcriptional regulation activity of PRC1.1.

Several ITD variants of the last BCOR exon have been described, resulting in an elongation of the protein between 22 and 60 amino acids. In several cases, short insertions of non-repeated nucleotide sequences were found at the end of duplication, as well as an internal tandem triplication in a single case [13,14,15,16,17]. ITDs involve the PUFD domain of the BCOR protein, which is crucial for the link with the RAWUL (RING finger and WD40-associated ubiquitin-like) domain of the PCGF1 protein, in the building of the PRC1.1 core [50]. To the best of our knowledge, studies on the impact of the ITD on the protein folding, assembly, and function of the PRC1.1 are missing. However, transcriptome analysis of CCSK, as well as of other BCOR-related tumors, allows us to speculate a certain degree of reduction in PRC1.1 activity and of its interaction with PRC2 [15].

Another element suggesting the hypothesis that the BCOR-ITD is associated with an impairment of the PRC1.1 function derives from the assessment of the DNA methylation state in CCSK. In fact, the genome of CCSK is hyper-methylated compared to that of other renal tumors [29,39]. This evidence could be related to a potential loss of function of KDM2B histone-demethylase, an enzymatic component of the PRC1.1 [51]. On the other hand, a possible reduction in KDM2B activity could be the consequence, rather than the cause, of the hyper-methylation of DNA, as it is electively recruited from the hypo-methylated CpG islands [52,53].

A third clue to this hypothesis can be found in the clinical and biological analogies between CCSK and the Endometrial Stromal Sarcomas bearing BCOR-ZC3H7B fusion protein. For these tumors, a clear loss of function of the PRC1.1 can be assumed, due to the important structural disruption of BCOR protein in the chimeric transcript and the absence of the Bcl-6 binding site (although PUFD is preserved) [54,55]. Finally, the lower activity of PRCs in BCOR-ITD-positive CCSK could partially explain the over-expression of BCOR. In fact, it has been shown that PRC components are frequently subject to transcriptional self-regulation [56].

The pathway enrichment analysis on the CCSK samples of this study found to be significantly up-regulated several protein coding genes related to MAPK signaling (previously associated with CCSK pathogenesis) [32,34,35,36]. FGF3, VEGFA, SPP1, ADM, and JUND were identified by differential expression analysis as genes of interest to justify the tendency to metastasize. FGF3 and VEGFA were also found over-expressed in metastatic CCSK compared to WT. FGF3, VEGFA, SPP1, and ADM are genes involved in many different functions, but they all have a role in cell proliferation and neo-angiogenesis. In fact, their over-expression has already been associated in multiple studies with the acquisition of the malignant phenotype.

Vascular endothelial growth factor was identified as an essential mitogen for the endothelial cells, with the ability to induce physiological and pathological angiogenesis and to promote vascular hyper-permeability [57,58]. VEGFA over-expression and its autocrine signaling are generally distinctive features of the most aggressive and poorly differentiated tumors [57,59]. VEGFA itself seems to act on tumor cells promoting the maintenance of an undifferentiated state [57], while its over-expression has already been related to CCSK [34,35,39,40].

Osteopontin (OPN) is a chemokine-like sialoprotein also known as bone sialoprotein 1 or secreted phosphoprotein type 1 (SPP1). The high expression of SPP1 is known to contribute to tumor progression by promoting neoplastic cells’ migration through cytoskeletal reorganization and homing of metastases to the bone matrix, contributing to cell proliferation by inhibiting apoptosis through activation of the Akt pathway and inducing neo-angiogenesis [60,61,62,63]. Moreover, SPP1 and VEGFA are frequently co-expressed, and it seems that the pathways of both molecules can induce the expression of the other one [64].

Adrenomedullin (ADM) is a small circulating peptide produced and secreted by tumor cells, but also by endothelial cells, macrophages, mast cells, and vascular smooth muscle cells. ADM secretion is mostly stimulated by oxidative stress, inflammatory stimuli, and hypoxia through trans-activation of HIF-1 (hypoxia-inducible factor 1), similar to VEGFA [65,66]. ADM acts as a powerful angiogenic agent [67,68] and its expression and plasmatic levels have been correlated to many aspects of tumor progression [69].

The *FGF3* gene, also known as int-2, was originally identified in mice as a site of murine breast cancer virus insertion, resulting in transcriptional activation of the gene and oncogenesis [70]. In humans, the *FGF3* gene belongs to the subfamily of FGF homologous to *FGF7*, and the encoded protein acts as paracrine growth factor with greater affinity for FGFR1-2 splicing variants IIIb [71]. The FGF family consists of secreted signal peptides, binding to the receptors with different affinities, as well as of proteins not associated with cell signaling, called intracellular FGFs (iFGFs). The secreted FGFs are almost ubiquitously expressed and activate a series of intracellular signaling pathways, including the MAPK and PI3K/Akt pathways [71,72,73,74]. They have essential roles both in early stages of embryonic development and in adults, where they act as homeostatic factors for tissue repair, regeneration, neo-angiogenesis, and metabolism [74].

Germline gain of function mutations, amplifications, and fusions of FGF genes can lead to abnormal morphogenesis or cause different types of cancer. These mutations can play an oncogenic role (by promoting cell survival and proliferation), enhance neoplastic progression (by stimulating angiogenesis or the acquisition of invasiveness), or provide the tumor with pathways to escape from targeted drugs [71,72,75,76]. Over-expression of FGF ligands is commonly observed in tumors, and over-expression of several ligands of the FGF family (FGF9/13/19) has also been observed in CCSK [34].

FGF3 expression is observed in human embryonic tissues but is usually not detectable in normal adult tissues. FGF3 over-expression has been detected in various tumor types, frequently associated with metastatic evolution and disease progression [72,77,78,79,80,81,82,83]. Interestingly, 55% of Kaposi’s Sarcoma, an endothelial tumor [84], over-express FGF3, thus highlighting the role of FGF3 in angiogenesis [85].

In CCSK, it is unclear whether the increased level of FGF3 transcript found in samples of metastatic tumors is directly related to an increased production by neoplastic cells or whether this is due to the effect of non-neoplastic stromal cells associated with the tumor, possibly stimulated by neoplastic cells through other factors. In any case, the over-expression of FGF3 could lead to positive feedback loops with a pro-proliferating and pro-angiogenic effect. These loops could be either paracrine, involving both stromal and tumor cells, or autocrine, involving only tumor cells, and could certainly help tumor progression of metastatic cases. Indeed, we showed that FGF3 is able to promote cell migration, with the highest migration induction on cells carrying the BCOR-ITD.

Altogether, the genes up-regulated in metastatic CCSK are suggestive of a global activation of the hypoxia and angiogenesis pathways, known to play a role in the progression and malignancy of human tumors. Indeed, FGF3, VEGF, and ADM are all potent inducers of neo-angiogenesis, while VEGF and ADM are both known HIF1-alpha target genes [86], raising the hypothesis that this whole molecular signature could be subtended by the activation of the hypoxic pathway. Overall, the data reported in this study suggest that the metastatic spread of BCOR-ITD-positive CCSK can be promoted by specific changes in the gene expression profile that enhance the malignant features of cancer cells and whose activity can in principle be counteracted by specific target pharmacological inhibition.

## 4. Materials and Methods

### 4.1. Patients and Tumor Samples

The analyzed case series consists of 9 patients affected by CCSK enrolled in the TW AIEOP 2003 protocol between January 2003 and May 2015. The histological diagnosis was performed by analysis of fresh frozen tissue (FF) snap-frozen in liquid nitrogen and stored at −80 °C or formalin-fixed paraffin-embedded (FFPE) specimens of CCSK collected during the surgical operation or open biopsy and subsequently revisioned and confirmed by the National Reference Centre for diagnosis of renal tumors in pediatric age. Three patients had metastases at diagnosis (CCSK 3-5-9). No genito-urinary anomalies or syndromic patterns were found in any of the patients. Patients’ characteristics are listed in Table 1.

For all patients, whole-exome sequencing (WES) was performed on all biological tumor samples and matched with peripheral blood samples or kidney samples, if available, in order to exclude germinal mutations. Whole-transcriptome sequencing (WTS) was performed in 8 cases. BCOR mutational status was assessed by Sanger sequencing and WTS. In order to further characterize non-metastatic and metastatic CCSKs, we compared their molecular analyses and profiling to 5 WT samples. Samples’ characteristics are listed in Table 2. This study was approved by the local institutional ethical committee of S. Orsola-Malpighi hospital.

### 4.2. WTS and WES

WTS data were already produced and published [14], while for WES analysis, genomic DNA was extracted from fresh frozen tumor specimens and from matched PB with QiAmp DNA mini kit (Qiagen) or with QiAmp DNA micro kit (Qiagen, Hilden, Germany) if the tumor sample was from FFPE block. Libraries were synthesized with Nextera Rapid Capture Exome Kit (Illumina, San Diego, CA, USA) following the manufacturer’s recommendations. Briefly, genomic DNA (50 ng for fresh frozen and 100 ng for FFPE samples) was tagged and fragmented by the Nextera transposome technique to an average library size of 290 bp. DNA libraries were then denatured to single-stranded DNA and hybridized to biotin-labeled 80 mer probes designed to enrich 214,126 targeted exonic regions, then eluted from magnetic beads and amplified.

WES libraries were quality-checked and sized with Agilent DNA 7500 chips on the Bioanalyzer 2100 (Agilent Technologies, Santa Clara, CA, USA), then quantified using a fluorometric assay (Quant-iT PicoGreen Assay, Life Technologies, Carlsbad, CA, USA). Paired-end libraries (12 pmol/L) were amplified and ligated to the flowcell by bridge PCR and sequenced at 2 × 100 bp using Illumina Sequencing by synthesis (SBS) technology.

### 4.3. Bioinformatic Analysis

After demultiplexing and FASTQ generation (both steps performed with bcltofastq function developed by Illumina), the paired-end reads were trimmed using AdapterRemoval (https://github.com/MikkelSchubert/adapterremoval (accessed on 3 March 2015)) with the aim of removing stretches of low-quality bases (<Q10) and Truseq/Nextera rapid capture adapters present in the sequences. The paired-end reads were then aligned on human reference genome HG19 (http://www.genome.ucsc.edu/ (accessed on 5 March 2015)) and analyzed with two different pipelines for WTS and WES data.

Sequences coming from RNA-seq were mapped with the algorithms TopHat/BowTie [87] and the PCR and optical duplicates were removed with the function rmdup of Samtools (http://samtools.sourceforge.net (accessed on 6 March 2015)). Gene expression profiling analysis was carried out first by adopting the function htseq-count (Python package Htseq: http://www.huber.embl.de/HTSeq/doc/overview.html (accessed on 23 March 2015)) to quantify the number of reads mapped on genes included in the Ensembl release 72 annotation features (http://www.ensembl.org (accessed on 25 March 2015)). Second, the evaluation of differentially expressed genes was performed with the R-Bioconductor package edgeR and limma (https://bioconductor.org/ (accessed on 7 April 2015)), respectively, to normalize and to compute the statistical analysis of differential gene expression.

Principal component analysis of gene expression profiling was performed with the function prcomp from stats R packages (https://www.r-project.org (accessed on 14 April 2015)), while Multiple Experiment Viewer (http://mev.tm4.org (accessed on 13 May 2015)) was adopted to the supervised hierarchical clustering using the Manhattan distance and the average linkage method. In order to identify the pathways overrepresented, we performed a gene set enrichment analysis with the WEB-based GEne SeT AnaLysis Toolkit (http://www.webgestalt.org (accessed on 22 May 2015)) using as a priori gene sets the KEGG pathways database. DeFuse (http://compbio.bccrc.ca/software/defuse/ (accessed on 4 June 2015)), ChimeraScan (https://code-google-com.ezproxy.unibo.it/archive/p/chimerascan/ (accessed on 5 June 2015)), Tophatfusion (https://ccb.jhu.edu/software/tophat/fusion_index.shtml (accessed on 5 June 2015)), and FusionMap (http://www.arrayserver.com/wiki/index.php?title=FusionMap (accessed on 12 June 2015)) methods were used to detect chimeric transcripts from RNA-seq data.

Data from WES were mapped with Burrows–Wheeler Aligner with the default setting [88]; the PCR and optical duplicates were removed as previously described for the RNA-seq, the Genome Analysis Toolkit (https://software.broadinstitute.org/gatk (accessed on 25 March 2015)) was used to locally realign, recalibrate, and call the Ins/del variants, while point mutations were identified with the algorithm Mutect (https://www.broadinstitute.org/cancer/cga/mutect (accessed on 13 July 2015)). Single-nucleotide variants (SNVs) and ins/del were annotated with a gene and protein alteration using Annovar (http://annovar.openbioinformatics.org (accessed on 15 July 2015)); nonsynonymous and nonsense SNVs, frameshift/non-frameshift Indels, and splice-site mutations were selected with a threshold read depth ≥15× and a variant allele frequency ≥0.2. All the variants were filtered in order to select novel or rare events (frequency in the population <1%), based on the database of human variability dbSNP (http://www-ncbi-nlm-nih-gov.ezproxy.unibo.it/SNP (accessed on 20 July 2015)), 1000 Genomes (http://www.1000genomes.org (accessed on 22 July 2015)), ExAC (http://exac.broadinstitute.org (accessed on 21 July 2015)), and EVS (http://evs.gs.washington.edu/EVS (accessed on 20 July 2015)). In-depth evaluation of high-confidence somatic variants was performed by verifying the presence of an alternate allele on the normal counterpart and manually visualizing each variation with the tview function of Samtools. Potential candidate drivers were highlighted considering the Catalog of Somatic Mutations in Cancer (http://cancer.sanger.ac.uk/cosmic (accessed on 25 July 2015)), pointing out the Cancer Gene Census set, and predicting the effect of the mutations on protein structure and function with SNPeff [89].

Moreover, based on WES data, the analysis of amplifications and large deletions was performed making a consensus between Control FREEC (http://boevalab.com/FREEC (accessed on 15 March 2016)) and ADTEX (http://adtex.sourceforge.net (accessed on 18 March 2016)) with paired tumor/matched normal samples. Furthermore, a filtering procedure was applied considering the uncertainty value given by Control FREEC (<80%) and the polymorphic copy-number variants from the Database of Human Genomic Variants (http://dgv.tcag.ca/dgv/app/home (accessed on 29 April 2016)). 

For germline variants’ prioritization, all rare (MAF  <  0.01) alterations occurring on the known cancer-related genes were considered. Moreover, variants with an evident effect on the protein (nonsense and splicing mutations or frameshift ins/del) were prioritized and manually annotated using HGMD and ClinVar database and with the literature (https://www.ncbi.nlm.nih.gov/clinvar/ (accessed on 6 April 2016)).

### 4.4. Sanger Sequencing

Sequencing of the DNA extracted from tumors and matched peripheral blood samples was performed to validate candidate mutations. Specific PCR assay for the amplification and sequencing of selected genes was designed with Primer Express 3.0 Software (Applied Biosystems, Monza, Italy). PCR products were purified with the Qiaquick PCR purification kit (Qiagen) and sequenced on both strands using the Big Dye Terminator v1.1 Cycle Sequencing kit (Applied Biosystems). Sanger Sequencing was performed on ABI 3730 Genetic Analyzer (Applied Biosystems).

### 4.5. Quantitative PCR

Total RNA was extracted from fresh frozen tissues using the RNeasy spin-column method (Qiagen). RNA was reverse transcribed to cDNA using the Transcriptor First-Strand cDNA Synthesis Kit (Life Technologies) with oligo dT primers. qPCR amplification of genes of interest was performed with real-time LightCycler 480 instrument (Roche). Fold-change was estimated by DDCt method, using GAPDH and GUSB as housekeeping. Significance (*p*-value) was estimated with the Student’s *t*-test.

Primers used were: SPP1_FW 5′-TTTGCCTCCTAGGCATCACC-3′ and SPP1_RV 5′-GCTTCTGAGATGGGTCAGGG-3′, VEGFA _FW 5′- TGAACTTTCTGCTGTCTTGGGT-3′ and VEGFA_RV 5′-ATGTCCACCAGGGTCTCGAT-3′, JUND_FW 5′- CTCAAGGACGAGCCACAGA-3′ and JUND_RV 5′-CAGCTCCGTGTTCTGACTCTT-3′, FGF3_FW 5′-GGGACGACTCTATGCTTCGG-3′ and FGF3_RV 5′-CAGGGAGGACTTCTGTGTGC-3′, ADM_FW 5′-ATGTCGCGTCGGAGTTTCG-3′, and ADM_RV 5′-GTTGTTCATGCTCTGGCGGTA-3′.

### 4.6. Cell Lines and Gene Editing

To reproduce the oncogenic model induced by BCOR, we designed a gene editing approach through Crispr/Cas9 in HEK-293 embryonal renal cells by co-transfection of a donor vector (pGL3basic, Addgene) with a left homology arm including a portion of intron 14, the full exon 15 carrying the ITD-2 cloned from a CCSK tumor sample, P2A linker upstream of a GFP recombination selection marker, and a right homology arm consisting of BCOR 3′UTR and a sgRNA vector derived by inserting the spacer sequence specific for BCOR last exon in a PX459Puro plasmid carrying the Cas9 sequence.

HEK-293 cells were grown in DMEM with 10% FBS (Euroclone), 20 µM L-Glutamine, and 1% Penicillin–Streptomycin mix (Gibco) and splitted every 4 days with trypsin 1X (Gibco). The day prior to the transfection, 5 × 10^5^ cells were seeded in a 6-well plate (Corning). Different wells were transfected with the mixture of the guide vector (gRNA) with the donor vector, using Lipofectamine 2000 (Invitrogen) as the transfecting reagent. After 24 h, transfected cells were selected for the following 48 h by adding Puromycin to the media at a final concentration of 3 µg/mL. The surviving cells were then sorted for GFP expression and expanded in 6-well plates before splitting them using serial dilutions in 96-well plates at a concentration of 0.5 cells/well. After 48–72 h the wells were inspected, and the single-cell clones were expanded and evaluated for GFP expression by flow cytometry.

PCR analysis on genomic DNA extracted from the clones with highest GFP expression revealed the presence of the BCOR-ITD insert in homozygosis. The primers used for PCR screening were: BCOR_ex15 Fw_5′-CCATTGCAGAGGCAGAATTTTA-3′, BCOR_ex15 Rev 5′- CTGTACATGGTGGGTCCAGCT-3′.

To obtain the scramble clone, cells were transfected with a mixture of the guide vector and an unspecific scramble vector. After 24 h, transfected cells were selected for the following 48 h by adding Puromycin to the media at a final concentration of 3 µg/mL. The surviving cells were harvested and seeded in serial dilutions to obtain single-cell colonies.

### 4.7. Flow Cytometry

For intracellular staining, cells were harvested, washed in PBS, then fixed and permeabilized in ice-cold 90% methanol overnight. Then, fixed cells were centrifuged, washed in PBS supplemented with 4% FBS, and incubated with primary rabbit anti-BCOR antibody (ab135801, Abcam, Cambridge, UK). After one-hour, cells were centrifuged and incubated with FITC-conjugated goat-anti-rabbit secondary antibody and analyzed through the FACSCanto-II flow cytometer (BD Biosciences, Franklin Lakes, NJ, USA). Flow cytometry data were analyzed with the FlowJo software, Version 10.8 (FlowJo, Ashland, OR, USA).

### 4.8. Migration Assay

BCOR-ITD and SCR clones were both splitted 1:1 in a new flask 24 h before the experiment. The transwells (Corning, with 8 µm pores) were coated with 2.5% Matrigel in DMEM and incubated at 37 °C for at least 2 h. Just before the seeding, the excessive media were removed and replaced with DMEM 1% FBS inside the transwell insert. The cells were detached and seeded at a concentration of 5 × 10^4^ cells/insert, then the inserts were placed in wells containing DMEM 20% FBS plus 12.5 ng/mL FGF3 (R&D).

After 48 h, all the cells inside the transwell were mechanically removed and the membranes were colored with Cell Stain Solution (Cell Biolab) for 15 min, then washed in distilled water and let to dry. The colored transwell inserts were put back in a dry plate and the migrated cells were counted on the microscope.

## 5. Conclusions

The present study, using next-generation sequencing approaches, confirmed CCSK as a tumor with a low mutational load probably not associated with syndromic patterns. In addition, in metastatic cases, the gene expression analysis showed the over-expression of genes potentially involved in neoplastic progression and metastatic processes, such as FGF3, VEGFA, SPP1, and ADM. The interest in these genes, if their de-regulation in metastatic CCSKs were to be confirmed in a larger series, is more remarkable because they are all associated with neo-angiogenesis and could represent therapeutic targets.

In fact, the use of various drugs against both VEGFs and FGFs signaling is already well-established, while anti-osteopontin and anti-adrenomedullin molecules represent promising new antineoplastic targeted therapies.

## Figures and Tables

**Table 1:** Patients’ characteristics. L, left; R, right; RT, radiotherapy; NR, no remission.

ID	Sex	Age (Months)	Side	Stage	Metastatic Site	Neoadjuvant Treatment	Surgery	Adjuvant Treatment	RT	Relapse
CCSK1	M	37	L	I	-	N	Y	Y	N	N
CCSK2	F	20	L	III	-	N	Y	Y	Y	N
CCSK3	M	27	R	IV	femur	Y	Y	Y	Y	Iliac wing
CCSK4	M	14	R	I	-	N	Y	Y	N	N
CCSK5	M	21	L	IV	lung	N	Y	Y	Y	N
CCSK6	M	27	L	I	-	N	Y	Y	N	N
CCSK7	F	17	L	II	-	Y	Y	Y	Y	N
CCSK8	F	25	R	II	-	Y	Y	Y	Y	N
CCSK9	M	22	R	IV	diffuse	Y	Y	Y	N	NR

**Table 2:** Samples’ features. FF, fresh frozen specimen; FFPE, formalin-fixed paraffin-embedded; PB, peripheral blood; WES, whole-exome sequencing; WTS, whole-transcriptome sequencing. ITD-1: c.5136_5225dup; ITD-2: c.5099_5212dup; ITD-3: c.5171_5266dup.

ID1	ITD-BCOR	Samples		WES		WTS
		Tumor	Normal	Tumor	Normal	
CCSK1	ITD-3	FF	PB	Y	Y	Y
CCSK2	ITD-1	FF	PB	Y	Y	Y
CCSK3	ITD-3	FF	NA	Y	N	Y
CCSK4	ITD-1	FF	PB	Y	Y	Y
CCSK5	ITD-3	FF	PB	Y	Y	Y
CCSK6	ITD-3	FF	NA	Y	N	Y
CCSK7	ITD-1	FF	FF	Y	Y	Y
CCSK8	ITD-2	FF	PB	Y	Y	Y
CCSK9	ITD-3	FFPE	PB	Y	Y	N

## Data Availability

The data presented in this study are available at the SRA repository https://www.ncbi.nlm.nih.gov/sra (accessed on 11 January 2023) with the Bioproject ID PRJNA924111.

## Supplementary Materials

The following supporting information can be downloaded at: https://www.mdpi.com/article/10.3390/ijms24043743/s1.
