# Supplementary material for: Molecular Signature of Biological Aggressiveness in Clear Cell Sarcoma of the Kidney (CCSK)
Source: Int J Mol Sci. 2023 Feb 13;24(4):3743. doi: 10.3390/ijms24043743 (PMC9964999; doi:10.3390/ijms24043743)
Supplement: Supplementary file 1 [file ijms-24-03743-s001.zip › ijms-2194511-supplementary.pdf]

Supplementary materials

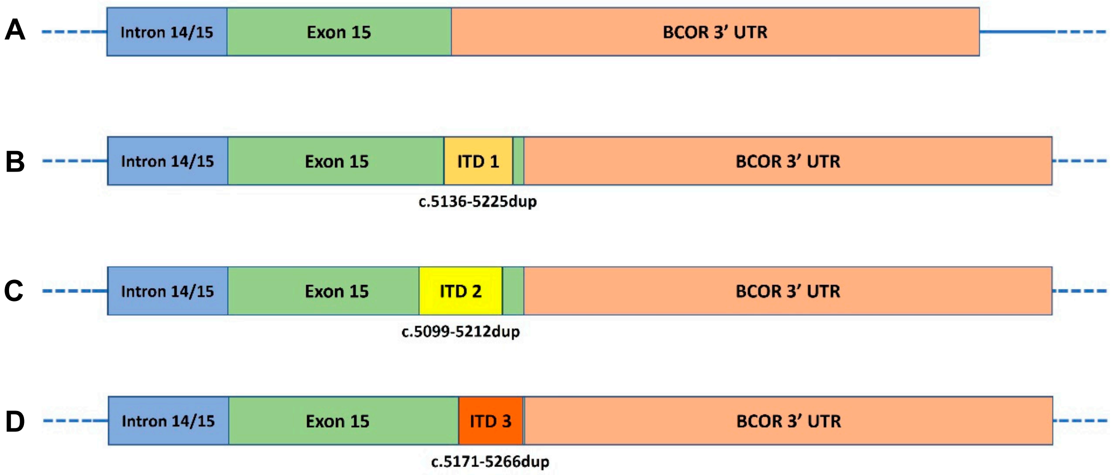

Figure S1. Representation of wild type BCOR last exon structure (**A**) and of ITD-1 (**B**), ITD-2 (**C**) and ITD-3 (**D**) position and dimension.

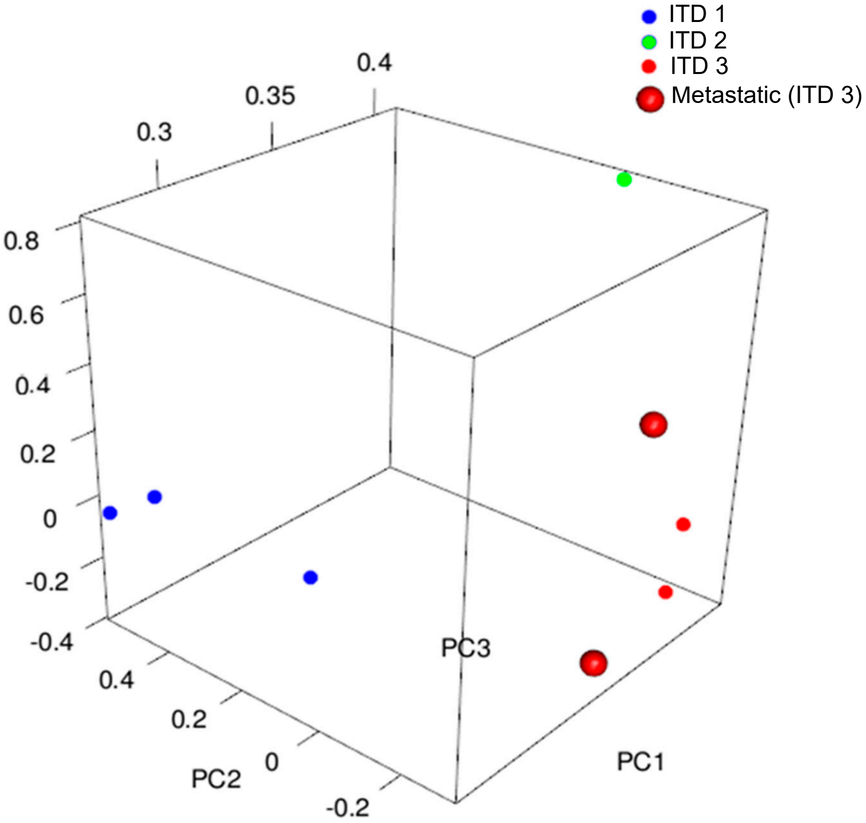

Figure S2. Principal component analysis (PCA) of the gene expression profiles of CCSK. ITD-1: c.5136_5225dup; ITD-2: c.5099_5212dup; ITD-3: c.5171_5266dup.

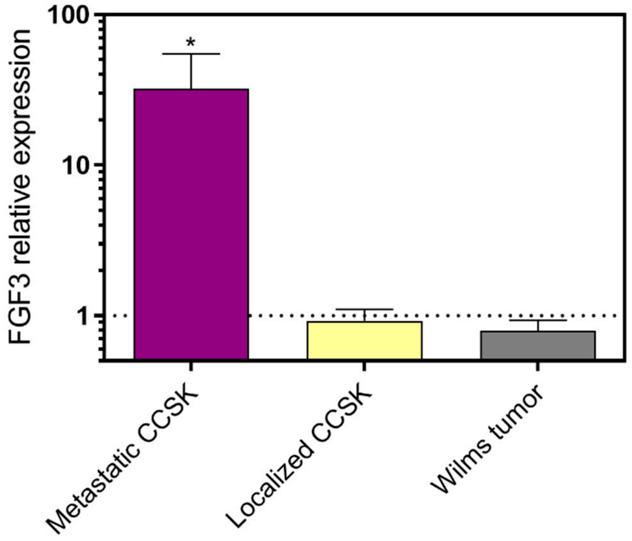

Figure S3. Expression of FGF3 in metastatic and localized CCSK, and in Wilms tumor samples measured by quantitative PCR (*, $p<0.05$).

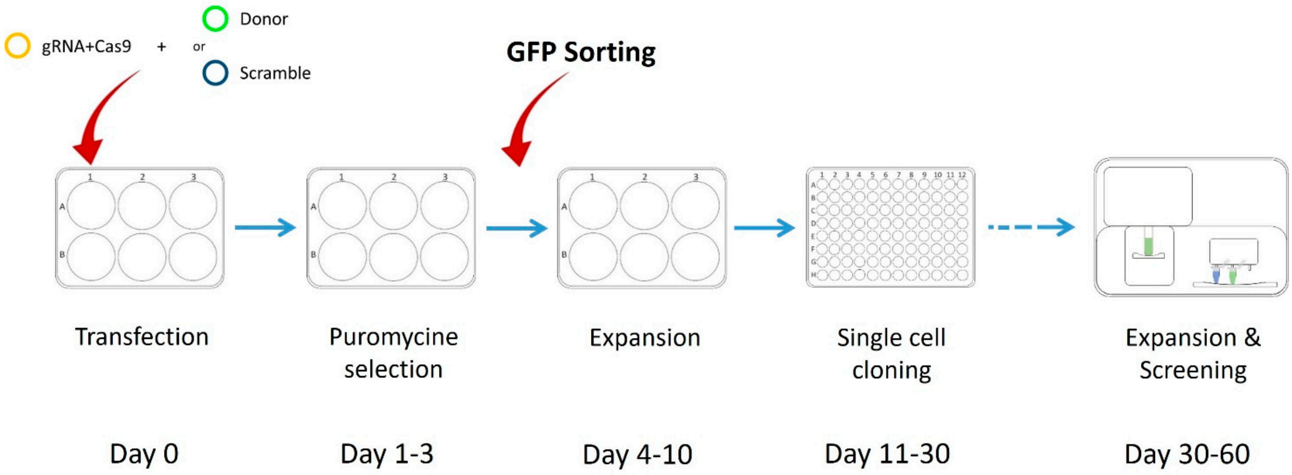

Figure S4. Experimental workflow of BCOR gene editing and clone selection. HEK-293 cells were transfected with a vector carrying the specific gRNA and the Cas9 ORF coupled with either the donor vector carrying the cloned ITD sequence or a scramble vector as control. After puromycin selection recombinant clones expressing the GFP sequence were selected by FACS sorting, amplified, cloned by limiting dilution and characterized by flow cytometry for GFP expression.

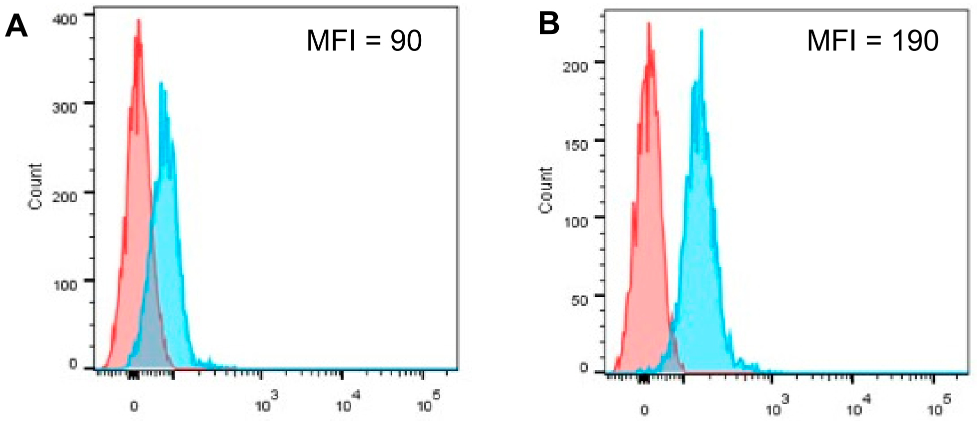

Figure S5. BCOR expression by flow cytometry in HEK-293 parental cell line (**A**) and BCOR-ITD edited clone (**B**). Blue profile: cells stained with primary anti-BCOR and FITC-conjugated anti-rabbit secondary antibody; Red profile: cells stained only with the FITC-conjugated secondary antibody.

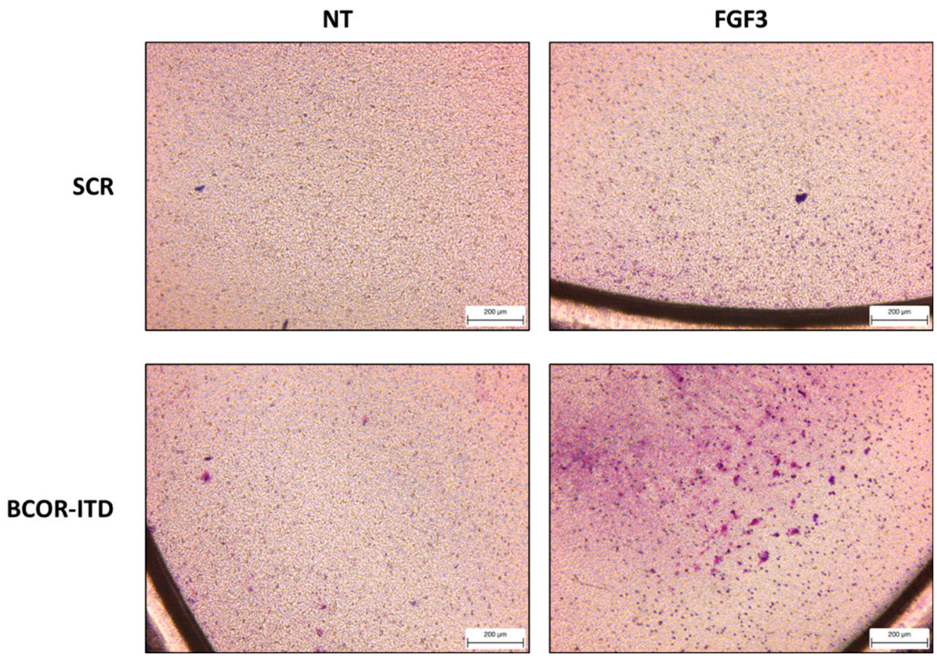

Figure S6. Pictures of the culture transwells showing the migrated cells colored with cell stain solution.
